# Promoting the Assessment of Frailty in the Clinical Approach to Cognitive Disorders

**DOI:** 10.3389/fnagi.2017.00036

**Published:** 2017-02-24

**Authors:** Marco Canevelli, Matteo Cesari, Francesca Remiddi, Alessandro Trebbastoni, Federica Quarata, Carlo Vico, Carlo de Lena, Giuseppe Bruno

**Affiliations:** ^1^Department of Neurology and Psychiatry, Sapienza University of RomeRome, Italy; ^2^Gérontopôle, Centre Hospitalier Universitaire de ToulouseToulouse, France; ^3^Institut du Vieillissement, Université de Toulouse III Paul SabatierToulouse, France

**Keywords:** frailty, cognitive disturbances, dementia, aging, holistic approach

## Introduction

The need of adapting healthcare systems (originally designed for younger patients with a lower burden of chronic conditions) to the specific needs of older individuals has been repeatedly evoked in light of the rapid aging of our populations (Beard et al., 2016; Cesari et al., 2016a). In this scenario, the so-called “frailty” condition has been attracting special interest. The concept of frailty is today no longer confined within the original geriatric boundaries, being currently implemented in various medical areas, from cardiology (Afilalo et al., 2009) to anesthesiology (Hubbard and Story, 2014), from infectious disease medicine (Brothers et al., 2014) to oncology (Balducci, 2013). It is indeed growingly considered as a crucial aspect for reshaping our healthcare systems, as requested by public health agencies (European Commission, 2015).

## Definition of frailty

Frailty is a geriatric condition characterized by the reduction of the individual's homeostatic reserves, leading to an increased vulnerability to endogenous and exogenous stressors (Morley et al., 2013). It is a strong predictor of health-related events (including functional decline, hospitalization, institutionalization, and death; Clegg et al., 2013). It is considered as resulting from the interaction between the progressive age-related decline in physiologic systems and chronic diseases and conditions, consequently leading to decreased functional reserve capacities (Cesari et al., 2016a).

Although there is a large agreement around the theoretical definition of frailty, multiple instruments have been developed with the aim of capturing and objectivating such state of increased vulnerability. Consequently, the estimates of frailty prevalence are largely dependent on the adopted operational definitions and vary substantially across available studies (Collard et al., 2012; Theou et al., 2015).

## Implications of frailty in the approach to cognitive disorders

Despite its scientific and clinical relevance, the concept of frailty is still rarely adopted in neurological settings and, in particular, in the field of cognitive disorders (Canevelli et al., 2014). This is quite surprising if considering that cognitive skills and capacities are recognized as strong contributors to the individual's vulnerability and resiliency to stressors, and that cognitive symptoms and disturbances become highly prevalent within the aging process (Canevelli et al., 2014).

The frailty construct may have important implications in the routine clinical approach to subjects attending clinical centers dedicated to the management of cognitive disturbances (i.e., memory clinics). First, it may consent to properly and multidimensionally consider the overall health status of the aging individual, thus not neglecting those “non-neurological” aspects (from sleep disorders to depression, from nutritional deficiencies to social issues, from polypharmacy to multimorbidity) potentially causing or contributing to the onset and progression of cognitive symptoms (Canevelli et al., 2015). This broader and holistic viewpoint is today required by the complexity of age-related pathological conditions (as cognitive disorders are; Canevelli et al., 2015), which are determined by multiple, simultaneous, and interacting pathophysiological processes. Promoting such approach does not automatically imply lower importance to those aspects that have traditionally and virtuously characterized the neurological practice (i.e., the accurate study of the neuronal and brain processes underlying the cognitive disturbances). It simply means integrating pivotal information about the heterogeneous aging of the individual. At the same time, an improved discrimination of true neurodegenerative conditions from the disturbances caused by the disruption of the homeostatic balance (indirectly responsible for the cognitive impairment) will have immediate impact in the clinical and research strategies (Cesari and Canevelli, 2014). For example, the assessment of frailty may provide useful indications for better planning and implementing individualized preventive and/or therapeutic interventions as well as support a more accurate prevision of the cognitive disturbance trajectory (Robertson et al., 2013). Weighting the overall clinical burden of the individual may also help at better distributing resources and improving the allocation of care services (Cesari et al., 2016b). Or, filtering the recruitment of participants in a clinical trial with a frailty assessment will allow maximizing the effect size of the interventions by selecting more or less “pure” individuals presenting a certain condition of risk.

## Instruments for assessing frailty

To date, there are more than 60 instruments available in literature for measuring frailty (Buta et al., 2016). The majority of them have been designed as screening instruments. Therefore, they may serve for the initial risk stratification of populations (Cesari et al., 2014). Conversely, the Frailty Index [proposed and validated by Rockwood and colleagues in the Canadian Study of Health and Aging (CSHA); Rockwood et al., 2005] is inapplicable at the first contact with an individual, and it can only be generated after (or in parallel with) a comprehensive clinical assessment (Cesari et al., 2014).

The Frailty Index may open interesting and promising scenarios in the field of neurodegenerative diseases and cognitive disorders (Kelaiditi et al., 2016). This instrument has been designed on an arithmetical model of “deficit accumulation;” i.e., the more deficits a person has, the more that person will be frail (Mitnitski et al., 2001; Rockwood and Mitnitski, 2012). Thus, it might be considered as an objective marker of “biological aging.” The Frailty Index is composed by a checklist of non-predefined variables (i.e., deficits) constituted by symptoms, signs, diseases, disabilities, and laboratory findings. The Frailty Index is simply the ratio between deficits presented by the individual and the total number of deficits considered [thus providing a continuous measure of frailty ranging between 0 (absence of frailty) and 1 (maximum of frailty); Searle et al., 2008]. Estimates of risk have been reported to be robust when a minimum set of 50 heterogeneous and multidimensional variables are considered. However, shorter versions (as low as composed by 20 items) have also been used in the literature (Searle et al., 2008).

Following a standard procedure (Searle et al., 2008), a Frailty Index can be retrospectively computed from available databases, even if these were created for completely different purposes. It is only important that the deficits to be considered are age-related, multidimensional (thus referring to different domains of the individual's health status), associated with negative outcomes, and not saturate too early or too late (i.e., being too highly or lowly prevalent in most people by some age; Searle et al., 2008). Since the Frailty Index weights every composing item as 1/*n* (where *n* is the total of considered deficits), no specific sign, symptom, or condition will be able to affect/bias the final score. At the same time, one item will become relevant if associated with other (more or less related) signs, symptoms, and/or conditions. In other words, the issue of standardizing different items in different settings is here resolved through the standardization and harmonization of the approach to the biological phenomenon, by using a mathematical model. Finally, by including disabilities in its computation, the Frailty Index provides meaningful results in every individual, independently of age and functional status. This solves the issue of a possible ceiling effect present in many tools designed for measuring frailty.

The Frailty Index has demonstrated consistent characteristics across samples and settings (Clegg et al., 2013). It has shown to increase with age, be strongly associated with negative health outcomes, and report higher values in women than in men (Peña et al., 2014). Given its continuous nature, the Frailty Index is also particularly sensitive to modifications over time and avoids the introduction of arbitrary decisions for the choice of defining cut-points (Rockwood et al., 2007). Thus, it may constitute a useful tool for describing the overall health status of the individual and measure its trajectory over time (also to ascertain the effectiveness of the implemented interventions; Cesari et al., 2014).

In light of its properties, the Frailty Index seems more appropriate to be adopted in the approach to subjects with cognitive disturbances in comparison to other available instruments developed to assess frailty. Differently from other operationalizations, the index is not based on a fixed set of criteria but on a quantitative/mathematic model. Thus, it can be computed, even retrospectively, from existing datasets and available clinical information. In parallel, it does not require the adoption of specific tests (e.g., assessment of physical performance), tools (e.g., dynamometers), and *ad hoc* questionnaires, potentially resulting in costly and time-consuming procedures. Conversely, it can be directly implemented in the clinical practice without requiring changes in the routine/standard approach to patients. This aspect appears to be crucial in the context of cognitive disturbances, considering that cognitive deficits may impair an individual's ability to perform specific tests and/or provide consistent answers about perceived symptoms or behavior. Finally, being founded on objective criteria, the Frailty Index is less prone to generate biases arising from the modification of pre-existing questionnaires and tools, as observed for other operational definitions (Theou et al., 2015).

The Frailty Index has already been cross-sectionally evaluated in studies exploring cognitive outcomes. In particular, it was found to be associated with poorer global cognitive performance (Rockwood et al., 2007), greater impairment in specific cognitive domains [such as neurocognitive speed (Rolfson et al., 2013) and executive functioning (Langlois et al., 2012)], and higher dementia prevalence (Armstrong et al., 2010).

More recently, the Frailty Index has been introduced for predicting the incidence of negative outcomes in neurology. Song and colleagues calculated a Frailty Index composed by deficits not known to predict dementia (e.g., fractures, cough, skin, and dental problems…) in 7,239 cognitively healthy, community-dwelling older adults in the CSHA (Song et al., 2011). This tool resulted to be significantly associated with the incidence of Alzheimer's disease (AD) and dementia of all types over 5- and 10-year follow-up periods. These associations were confirmed even after adjusting for age and traditional risk factors. Interestingly, each accumulated deficit increased the odds ratio of dementia by 3.2%. The Frailty Index also significantly discriminated people with dementia from those who were cognitively healthy.

A Frailty Index has recently been retrospectively computed and adopted in the Impact of Cholinergic Treatment USe (ICTUS) study, enrolling 1,375 mild to moderate AD patients across 12 European countries. In a first post-hoc analysis, a 30-item Frailty Index resulted to be significantly associated with mortality and hospitalization after 2 years of follow-up, exhibiting a borderline significance also for the institutionalization endpoint (Kelaiditi et al., 2015). In the same cohort of patients, the Frailty Index was found to strongly predict the rate of cognitive decline as measured by the modifications of the Mini Mental State Examination (MMSE) and Alzheimer's Disease Assessment Scale–Cognitive subscale (ADAS-Cog) scores (Kelaiditi et al., 2016). In particular, the presence of additional item (equivalent to a 3.3% increase in the Frailty Index) was associated with a clinically meaningful worsening of cognitive performance as indicated by a decline of 2.63 and 6.96 points in the MMSE and ADAS-Cog scores after 1 year of follow-up. Along the same lines, in the CSHA and in the Honolulu-Asia Aging Study, cognitive changes (measured by Modified Mini Mental State Examination and by the Cognitive Abilities Screening Instrument, respectively) were significantly influenced by the baseline Frailty Index (Mitnitski et al., 2011a,b; Armstrong et al., 2016). Accordingly, frail people were less likely to show cognitive improvement or stabilization over time compared to non-frail participants.

Based on these considerations, the Memory Clinic of the “Sapienza” University of Rome (Italy) has recently decided to systematically implement the Frailty Index, building it from signs, symptoms, and conditions that are routinely checked in all the individuals referring to its service for evaluation of cognitive disturbances. Thus, its computation does not imply any modification of the daily clinical practice. The detailed list of deficits composing the instrument is presented in Table 1 just as an example, in order to underline how a Frailty Index can be created in every clinical setting.

**Table 1:** **Example of the 50 items composing the Frailty Index currently used at the Memory Clinic of the “Sapienza” University of Rome**.

**50-ITEM FRAILTY INDEX**
Osteoporosis
Hypertension
Arthritis
Gastric disorder
Intestinal disorder
Ischemic heart disease
Chronic heart failure
Arrhythmia
History of stroke
History of TIA
Diabetes
Renal failure
Thyroid disease
Cancer
Cirrhosis
Peripheral artery disease
COPD
Dyslipidemia
Falls
Vertigo
Balance disorders
Involuntary weight loss (≥4.5 kg in the last 6 months)
Obesity (BMI ≥ 30 kg/m^2^)
Underweight (BMI < 18.5 kg/m^2^)
Focal neurological signs
Parkinsonism
Peripheral neuropathy
Depression
Anxiety
Sleep disorders
Memory complaint
Language disturbances
Spatiotemporal disorientation
Hearing impairment
Urinary incontinence
Mobility disability (inability to walk 400 m)
Hep with drugs (IADL)
Help with shopping (IADL)
Help with money (IADL)
Help with telephone (IADL)
Help with transportation (IADL)
Help toileting (ADL)
Help eating (ADL)
Help dressing (ADL)
Help transferring (ADL)
Hemoglobin (<13.5 g/dL in males; <12.0 g/dL in females)
Creatinine (<0.6 mg/dL)
Albumin (<3.5 g/dL)
Folic acid (<2.7 ng/ml)
White blood cells (<4 × 10^3^/mm^3^)

## Conclusions

The assessment of frailty may significantly improve the clinical and research approach to cognitive disturbances, also facilitating a more balanced and holistic understanding of these complex and burdening problems. In this regard, further research is needed in order to disentangle the complex network of pathophysiological mechanisms potentially affecting both cognition and individual vulnerability/resiliency. The Frailty Index may respond to the need of adjusting standard instruments to the heterogeneous population of subjects with cognitive disturbances, adding a useful estimate of the individual's biological aging to the other traditional neurological and cognitive evaluations.

## Author contributions

MC and MCe conceived the article and wrote the manuscript. FR, AT, FQ, and CV participated in performing the literature review and writing the paper. Cd and GB contributed to the critical appraisal of the manuscript.

### Conflict of interest statement

MCe has received honoraria for presentations at scientific meetings and/or research fundings from Nestlé and Pfizer. He is involved in the coordination of an Innovative Medicines Initiative-funded project [including partners from the European Federation Pharmaceutical Industries and Associates (Sanofi, Novartis, Servier, GSK, Lilly)]. The other authors declare that the research was conducted in the absence of any commercial or financial relationships that could be construed as a potential conflict of interest.
